# Long‐term virologic responses to antiretroviral therapy among HIV‐positive patients entering adherence clubs in Khayelitsha, Cape Town, South Africa: a longitudinal analysis

**DOI:** 10.1002/jia2.25476

**Published:** 2020-05-14

**Authors:** Kathleen Kehoe, Andrew Boulle, Priscilla R Tsondai, Jonathan Euvrard, Mary Ann Davies, Morna Cornell

**Affiliations:** ^1^ Centre for Infectious Disease Epidemiology and Research School of Public Health and Family Medicine University of Cape Town Cape Town South Africa; ^2^ Khayelitsha ART Programme and Médecins Sans Frontières Cape Town South Africa; ^3^ Health Impact Assessment Provincial Government of the Western Cape Cape Town South Africa

**Keywords:** HIV, antiretroviral therapy, antiretroviral treatment, adherence clubs, virologic failure, elevated viral load, virologic responses, Sub‐Saharan Africa, differentiated service delivery models, viral load monitoring

## Abstract

**Introduction:**

In South Africa, an estimated 4.6 million people were accessing antiretroviral therapy (ART) in 2018. As universal Test and Treat is implemented, these numbers will continue to increase. Given the need for lifelong care for millions of individuals, differentiated service delivery models for ART services such as adherence clubs (ACs) for stable patients are required. In this study, we describe long‐term virologic outcomes of patients who have ever entered ACs in Khayelitsha, Cape Town.

**Methods:**

We included adult patients enrolled in ACs in Khayelitsha between January 2011 and December 2016 with a recorded viral load (VL) before enrolment. Risk factors for an elevated VL (VL >1000 copies/mL) and confirmed virologic failure (two consecutive VLs >1000 copies/mL one year apart) were estimated using Cox proportional hazards models. VL completeness over time was assessed.

**Results:**

Overall, 8058 patients were included in the analysis, contributing 16,047 person‐years of follow‐up from AC entry (median follow‐up time 1.7 years, interquartile range [IQR]:0.9 to 2.9). At AC entry, 74% were female, 46% were aged between 35 and 44 years, and the median duration on ART was 4.8 years (IQR: 3.0 to 7.2). Among patients virologically suppressed at AC entry (n = 8058), 7136 (89%) had a subsequent VL test, of which 441 (6%) experienced an elevated VL (median time from AC entry 363 days, IQR: 170 to 728). Older age (adjusted hazard ratio [aHR] 0.64, 95% confidence interval [CI] 0.46 to 0.88), more recent year of AC entry (aHR 0.76, 95% CI 0.68 to 0.84) and higher CD4 count (aHR 0.67, 95% CI 0.54 to 0.84) were protective against experiencing an elevated VL. Among patients with an elevated VL, 52% (150/291) with a repeat VL test subsequently experienced confirmed virologic failure in a median time of 112 days (IQR: 56 to 168). Frequency of VL testing was constant over time (82 to 85%), with over 90% of patients remaining virologically suppressed.

**Conclusions:**

This study demonstrates low prevalence of elevated VLs and confirmed virologic failure among patients who entered ACs. Although ACs were expanded rapidly, most patients were well monitored and remained stable, supporting the continued rollout of this model.

## 1 Introduction

In 2018, there were approximately 37.9 million people living with HIV (PLHIV), with nearly 23.3 million PLHIV accessing antiretroviral therapy (ART) [1]. HIV prevalence in South Africa was 13% (7.4 million) in 2018, with estimated ART coverage of 62% (4.6 million) [2]. In September 2016, South Africa adopted the World Health Organization universal Test and Treat strategy, substantially increasing the number of individuals eligible for lifelong ART services [3, 4].

This expansion of ART has major implications in terms of service delivery and costs. Differentiated service delivery (DSD) models for ART services are being explored to ensure PLHIV are retained and remain virologically suppressed in long‐term care. One such model, adherence clubs (ACs), reduces the burden on patients and on the healthcare system by decentralizing ART services for stable patients [5]. ACs are facilitated by lay healthcare workers and require less frequent medication collections and clinical consultations than the standard of care (SOC). A pilot study of ACs in Khayelitsha in 2007 reported decreased loss to follow‐up (LTFU) and virologic rebound and improved retention compared to SOC [5, 6]. Following the initial success of this model of care, the Cape Metro Health District adopted ACs in 2011 and by March 2015, 1308 ACs were operating at 55 of the 70 facilities [7]. The scale‐up increased the proportion of patients accessing ART services in ACs from 7% in 2011 to 41% by the end of 2016 [7, 8]. The AC model was adopted into national policy in 2016 and has subsequently been implemented in six other provinces (Gauteng, Limpopo, Mpumalanga, Eastern Cape, KwaZulu‐Natal and the Free State) [9, 10].

Given the rapid expansion of ACs, it is important to evaluate their effectiveness over time. The proportion of completed viral load (VL) tests at expected dates can be used as a proxy for the quality of care and provide insight into the effectiveness of ART programmes [11]. In assessments following the scale‐up of ACs, VLs were recorded in 88% of patients, with 97% of these virologically suppressed, 13 months before analysis closure [3].

To date, outcome assessments of ACs in the Western Cape have largely focused on virologic rebound, suppression, retention and LTFU [3, 5, 7]. To the best of our knowledge, confirmed virologic failure and longer‐term outcomes have not yet been described. In this study, we assessed the proportion and predictors of elevated VLs and confirmed virologic failure among adult patients who have ever entered an AC in Khayelitsha. We also described VL completeness and virologic suppression over time.

## 2 Methods

### 2.1 Setting

Khayelitsha is a peri‐urban township in Cape Town, with an estimated population of 500,000 [12]. Khayelitsha has the highest burden of HIV in the Western Cape and one of the highest in South Africa [13, 14]. In 2012, the prevalence of HIV among pregnant women was 34% in Khayelitsha compared with 30% nationally [14, 15]. Public‐sector ART services were available from 2001 through pilot projects conducted at three primary care clinics by Médecins Sans Frontiéres (MSF) and the Western Cape Government [12, 13, 16, 17]. In 2004 the national ART programme was launched; by 2017, almost 40,000 patients were receiving ART in the Khayelitsha sub‐district ART programme [8].

### 2.2 Adherence clubs

ACs were first piloted in Khayelitsha by MSF in 2007 and were subsequently scaled up in 2011 due to their success [6, 7]. The AC guidelines define patients as stable if they have one suppressed VL (<400 copies/mL) and have been on ART for a minimum of six months with no chronic illnesses requiring regular consultation [7, 18]. ACs consist of 25 to 30 stable patients who participate voluntarily and meet with a lay healthcare worker five times annually. During these visits, they are weighed, receive a two‐month supply of pre‐packaged ART in person, have a brief symptom screening and a group discussion [3, 7, 19]. Typically patients collect their pre‐packaged ART, however, they may send a buddy to collect their medication at every alternate AC meeting [3, 7]. Patients are required to have a VL assessment four months after joining an AC, and annually thereafter, which are performed in batches for each AC [18]. Ill, non‐adherent patients or patients with a VL ≥400 copies/mL are referred back to receive the SOC at a facility.

We did not have access to the ART regimens for this cohort, but according to guidelines at the time patients were commonly on Tenofovir (Tenofovir, Emtricitabine/Lamivudine and Efavirenz) or Abacavir (Abacavir, Lamivudine and Efavirenz) based first‐line ART regimens [4]. Patients were switched to second‐line ART regimens in the event of two separate VLs >1000 copies/mL. Patients failing Tenofovir‐based regimens would have been switched to Tenofovir, Zidovudine, Lamivudine/Emtricitabine and Lopinavir/Ritonavir or Zidovudine, Lamivudine and Lopinavir/Ritonavir. Patients failing Abacavir regimens would have been switched to Zidovudine, Lamivudine and Lopinavir/Ritonavir.

### 2.3 Data source

This study used routinely recorded, anonymized data contributed to the International epidemiology Databases to Evaluate AIDS Southern Africa (IeDEA‐SA) collaboration from the Khayelitsha ART programme from January 2011 to June 2017 [20].

### 2.4 Eligibility criteria

Patients who initiated ART from January 2004 onwards, enrolled into an AC in provincial clinics in Khayelitsha between January 2011 and December 2016, were aged 16 to 85 years and had a suppressed VL (VL <400 copies/mL) captured in the dataset before AC entry were eligible for inclusion. We chose the start date of January 2011 to allow long‐term virologic outcomes to be assessed since the Cape Metro Health District adopted this model of care into guidelines in 2011.

### 2.5 Outcomes

The primary outcomes of interest were the proportion of patients with i) an elevated VL and ii) confirmed virologic failure after AC entry. An elevated VL was defined as a VL >1000 copies/mL after AC entry [21]. Confirmed virologic failure was defined as two consecutive VLs >1000 copies/mL performed within a 12‐month window. Additionally, our outcome includes duration spent in an AC, which corresponds to the follow‐up time as time zero in the model was the date of enrolment into an AC. VL completeness was defined as the proportion of patients who had a VL test among those followed up for that time period. Secondary outcomes were to describe the proportion of patients with VL tests done and virally suppressed. Viral suppression was defined as a VL test <400 copies/mL.

### 2.6 Statistical analyses

We described characteristics at enrolment into an AC (sex, age, year of AC entry, duration on ART, CD4 count and facility) at AC entry using medians and interquartile ranges (IQR) for continuous variables and frequencies and proportions for categorical variables. We used a window of 15 months before AC entry to determine CD4 counts and VL tests at AC entry, given that testing is recommended 12‐monthly, but may occur at slightly longer intervals in practice.

We assessed predictors of an elevated VL and confirmed virologic failure using univariable and multivariable Cox proportional hazards models adjusted for characteristics at enrolment. Age, sex, CD4 count and duration on ART were included as categorical variables and year of AC entry was included as a continuous variable. Time zero for the survival analysis was time of enrolment into an AC. We included patients who had at least one VL test on file after AC entry in the model assessing an elevated VL. We restricted the model for confirmed virologic failure to patients who experienced an elevated VL after AC entry and had a repeat VL test within a year. We report crude and adjusted hazard ratios (HR) with 95% confidence intervals (CI). Covariates in the Cox proportional hazards model were chosen based on previously published literature. The three clinics included in this study are within 10 kilometres of each other, serving a similar population, and we do not believe the clinics are acting as a random effect. Censoring occurred at death, transfer to another facility, LTFU, at analysis closure (June 2017) or at the time when they experienced virologic failure. Patients were defined as LTFU if they had no contact with the facility in the six months following analysis closure until database closure (December 2017) [22]. We performed a sensitivity analysis to assess differential follow‐up time in an AC as a potential confounder. We restricted this analysis to patients who were followed up for 475 days (sum of the median time to an elevated VL and confirmed virologic failure) and recoded the outcomes in this time period to assess if year of AC entry was acting as a confounder.

VL completeness was assessed at 4, 16, 28 and 40 months for all eligible patients, in accordance with the AC guidelines. We used a 0 to 10 month window for the four‐month assessment for patients followed up for at least 10 months, and a 12‐month window around subsequent time points (16, 28 and 40 months) for patients followed up until the end of each window. Importantly, these windows were discrete and did not overlap. The proportion of patients followed up for each time period was assessed for VL tests and categorised into <400, 400 to 1000 and >1000 copies/mL. In the analysis restricted to patients who had experienced an elevated VL after AC entry, we used Kaplan‐Meier estimators to assess the proportion of patients that re‐suppressed through four years of follow‐up. Data were cleaned, coded and analysed using Stata version 15.1 (College Station, TX, USA).

### 2.7 Ethics

The Human Research Ethics Committee in the Faculty of Health Sciences at the University of Cape Town approved this study (HREC REF 264/2018). Participating sites in Khayelitsha had ethical approval from their institutional review board to contribute anonymized, individual‐level data to IeDEA‐SA. These data were not identifiable, therefore informed consent was not required.

## 3 Results

Of the 8413 eligible patients, 63 (0.7%) patients were excluded for the following reasons: missing gender (5), unable to link on patient identifier (58) and a VL ≥400 copies/mL at AC entry (292). Overall, 8058 patients were included in the analysis and followed up for 16047 person‐years from AC entry, with a median follow‐up time of 1.7 years (IQR 0.9 to 2.9).

Patients entering ACs were predominantly female (74%) and aged between 35 to 44 years (46%) (Table 1). Entry into ACs increased over time, with the majority of AC entry (62%) occurring in 2015 to 2016. The median duration on ART at AC entry was 4.8 years (IQR 3.0 to 7.2). The median CD4 count at AC entry was 531 cells/µL (IQR 401 to 684), with a large proportion of missing CD4 counts (40%) observed. The majority of patients attended ACs at clinic 3 (42%). At analysis closure, 91 patients (1%) had died, 288 patients (4%) had transferred care, 683 patients (8%) were classified as LTFU and 6996 (87%) were retained.

**Table 1:** Characteristics of patients entering adherence clubs in Khayelitsha

Patient characteristics	Total cohort (n = 8058)
Sex, n (%)	
Males	2066 (26)
Females	5992 (74)
Age (years), n (%)	
Median (IQR)	39 (34 to 45)
16 to 34	2498 (31)
35 to 44	3701 (46)
≥45	1859 (23)
Year of AC entry, n (%)	
2011 to 2012	466 (6)
2013 to 2014	2588 (32)
2015 to 2016	5004 (62)
Duration on ART (years) at AC entry, n (%)	
Median (IQR)	4.8 (3.0 to 7.2)
0 to 2	2056 (26)
3 to 4	2131 (26)
≥5	3871 (48)
CD4 count (cells/µL) at AC entry, n (%)	
Median (IQR)	531 (401 to 684)
<500	2133 (27)
≥500	2681 (33)
Missing	3244 (40)
Facility	
Clinic 1	1676 (21)
Clinic 2	2951 (37)
Clinic 3	3431 (42)

**Figure 1:**
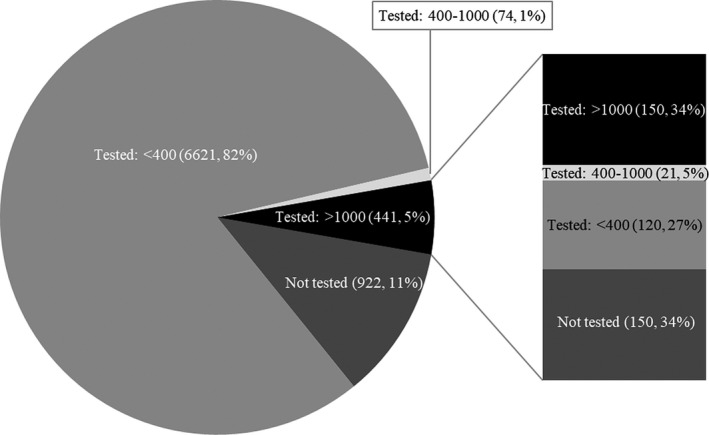
Viral load (VL) testing among suppressed patients who entered an adherence club (n = 8058). The pie chart represents the first viral load after adherence club entry. The stacked bar chart represents the second viral load test among patients with an elevated viral load (n = 441) at their first test after adherence club entry.

In the 8058 patients virally suppressed at AC entry, 11% (n = 922) did not have any subsequent VL tests done after AC entry (Figure 1). These 922 patients were predominantly female (74%), aged 35 to 44 years (41%), on ART for ≥5 years (40%), mostly had a missing CD4 count at AC entry (55%) and entered ACs in 2015 to 2016 (74%). Among those with a subsequent VL test done (n = 7136), the median time from AC entry to an elevated VL test was 363 days (IQR 170 to 728). The VL tests after AC entry were as followed: 93% (6621/7136) maintained virologic suppression, 1% (74/7136) had a VL between 400 to 1000 copies/mL and 6% (441/7136) experienced an elevated VL>1000 copies/mL. In sensitivity analyses, the proportion of patients with an elevated VL was 9% and 11% when we assumed that 50% and 100% of patients LTFU developed the outcome respectively.

In the univariable analysis of patients virally suppressed at AC entry with subsequent VL tests performed, the risk of experiencing an elevated VL decreased with age (HR 0.71, 95% CI 0.55 to 0.93, ≥45 years versus 16 to 34 years) and was decreased for patients who had entered ACs in more recent years (HR for each increasing year 0.84, 95% CI 0.78 to 0.92) (Table 2, Model 1). In the multivariable analysis, the protective effect of older age group persisted with patients aged ≥45 years being 34% less likely to have an elevated VL compared to those aged 16 to 34 years (adjusted HR (aHR) 0.66, 95% CI 0.50 to 0.87) (Table 2, Model 2). In the adjusted model, patients entering ACs in more recent years still had a reduced risk of experiencing an elevated VL (aHR for each increasing year 0.85, 95% CI 0.78 to 0.92). Similar results were obtained when CD4 count was included in the model (Table 2, Model 3). Compared with patients who had a CD4 count <500 cells/µL, those entering ACs with a CD4 count ≥500 cells/µL had a 33% reduced risk of an elevated VL (aHR 0.67, 95% CI 0.54 to 0.84). Duration on ART had no effect in any of the above models.

**Table 2:** Among virologically suppressed patients at adherence club entry, crude and adjusted associations with an elevated viral load and confirmed virologic failure, after entry into an adherence club

	First elevated viral load			Confirmed virologic failure		
	Model 1 HR (95% CI)	Model 2 aHR (95% CI) (n = 7136)	Model 3 aHR (95% CI) (n = 4395)	Model 1 HR (95% CI)	Model 2 aHR (95% CI) (n = 291)	Model 3 aHR (95% CI) (n = 219)
Sex						
Male	1.07 (0.86 to 1.32)	1.16 (0.93 to 1.44)	1.11 (0.85 to 1.44)	1.18 (0.82 to 1.69)	1.29 (0.88 to 1.90)	1.29 (0.81 to 2.06)
Age group (years)						
16 to 34	1.0	1.0	1.0	1.0	1.0	1.0
35 to 44	0.86 (0.69 to 1.06)	0.80 (0.64 to 1.00)	0.70 (0.54 to 0.91)	1.13 (0.79 to 1.60)	0.96 (0.66 to 1.39)	0.96 (0.62 to 1.48)
≥45	0.71 (0.55 to 0.93)	0.66 (0.50 to 0.87)	0.64 (0.46 to 0.88)	0.74 (0.45 to 1.19)	0.61 (0.37 to 1.02)	0.52 (0.28 to 0.94)
Year of AC entry	0.84 (0.78 to 0.92)	0.85 (0.78 to 0.92)	0.76 (0.68 to 0.84)	1.37 (1.18 to 1.59)	1.36 (1.16 to 1.58)	1.14 (0.93 to 1.39)
Duration on ART at AC entry (years)						
0 to 2	1.0	1.0	1.0	1.0	1.0	1.0
3 to 4	1.26 (0.96 to 1.65)	1.25 (0.95 to 1.65)	1.23 (0.89 to 1.70)	0.95 (0.58 to 1.54)	1.02 (0.62 to 1.66)	0.94 (0.52 to 1.70)
≥5	1.18 (0.92 to 1.51)	1.27 (0.98 to 1.64)	1.25 (0.92 to 1.70)	1.25 (0.81 to 1.95)	1.34 (0.84 to 2.11)	1.51 (0.88 to 2.60)
CD4 count at AC entry (cells/µL)						
<500	1.0	–	1.0	1.0	–	1.0
≥500	0.69 (0.55 to 0.85)	–	0.67 (0.54 to 0.84)	0.79 (0.54 to 1.15)	–	0.75 (0.50 to 1.11)

Model 1: univariable analysis; model 2: multivariable analysis including sex, age, year of AC entry and duration on ART at AC entry; model 3: model 2 including CD4 count at AC entry. For each outcome, only patients with a viral load on file were included in the model.

Of the 441 patients with an elevated VL, 150 (34%) patients did not receive a repeat VL test within a year (Figure 1). Among the 291 patients that experienced an elevated VL and had a repeat VL test done, 41% (120/291) successfully re‐suppressed, 7% (21/291) had a VL between 400 to 1000 copies/mL and 52% (150/291) still had an elevated VL >1000 copies/mL and were confirmed to have virologic failure. The overall prevalence of confirmed virologic failure in this cohort was 2% cross‐sectionally at the end of follow‐up. The median time from an elevated VL test to a confirmed virologic failure test was 112 days (IQR 56 to 168). In sensitivity analyses, the proportion of patients who subsequently experienced confirmed virologic failure was 54% and 55% when we assumed that 50% and 100% of patients LTFU developed the outcome respectively.

Year of AC entry predicted the risk of confirmed virologic failure. Patients who entered an AC in the more recent years had an increased risk of confirmed virologic failure, in both the univariable (HR for each increasing year 1.37, 95% CI 1.18 to 1.59) and multivariable models (aHR for each increasing year 1.36, 95% CI 1.16 to 1.58) (Table 2, Models 1 & 2). However, this effect was attenuated after adjusting for CD4 count at AC entry (Table 2, Model 3). In the model adjusted for CD4 count, patients in the older age group had a decreased risk of confirmed virologic failure (aHR 0.52, 95% CI 0.28 to 0.94, ≥45 years vs. 16 to 34 years) (Table 2, Model 3).

In a sensitivity analysis, we restricted the window for a suppressed VL to six months before AC entry (n = 5367, 64% of the cohort) in accordance with AC guidelines. Using the more stringent criteria, similar predictors for both an elevated VL and confirmed virologic failure were identified (Table S1). Patients with a longer duration on ART had an increased risk for confirmed virologic failure (aHR 2.53, 95% CI 1.14 to 5.60, ≥5 years vs. 0 to 2 years). We reported the multivariable analysis including CD4 count at AC entry, as the estimates were similar with and without CD4 count. Additionally, we looked at the effect of year of AC entry among patients who were only followed up for 475 days (Table S2). We found similar estimates, specifically for year of AC entry when assessing an elevated VL (aHR for each increasing year 0.73, 95% CI 0.63 to 0.84).

Among patients with sufficient follow‐up for each time point, the completeness of VL tests remained similar over time: 82% at 4 months and 83% at 40 months with high proportions of viral suppression observed (97% at 4 months and 93% at 40 months) (Table 3). The proportion of patients entering ACs virally suppressed and ever experiencing a VL >1000 copies/mL increased by 4 percentage points over time on ART (2% at 4 months and 6% at 40 months).

**Table 3:** Viral load completeness at 4, 16, 28 and 40 months after entry into an adherence club among patients who were suppressed at adherence club entry

Follow‐up time (months)	4	16	28	40
Number of patients, n	6547	3856	2170	1061
Viral load tests completed, n (%)	5340 (82)	3171 (82)	1841 (85)	884 (83)
Viral load (copies/mL), n (%)				
<400	5159 (97)	2997 (95)	1730 (94)	822 (93)
400 to 1000	40 (1)	52 (1)	26 (1)	8 (1)
>1000	141 (2)	122 (4)	85 (5)	54 (6)

**Figure 2:**
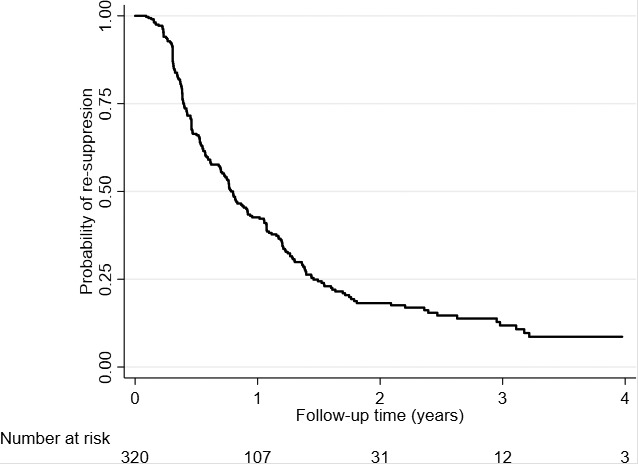
Probability of re‐suppression among patients who experienced an elevated viral load (VL) and had a subsequent viral load test.

Among patients who experienced an elevated VL after AC entry (n = 441), 121 (27%) did not receive a VL test through four years of follow‐up. Of the 320 patients who did receive a test, the overall proportion of patients who re‐suppressed was 73% (n = 234). Within the first year after experiencing an elevated VL, 52% of patients had re‐suppressed (Figure 2).

## 4 Discussion

This study demonstrated that the prevalence of elevated VLs and confirmed virologic failure was low for patients entering ACs since scale‐up of the model in Khayelitsha. The majority of patients were retained and remained virally suppressed over time. Virologic monitoring was generally consistent with approximately 80% completeness over time, although confirmatory tests were not performed on approximately one third of patients within a year experiencing an elevated VL.

Poorer health outcomes may have been expected given the rapid expansion of ACs. However, the overall prevalence of patients with confirmed virologic failure was 2% from January 2011 to June 2017. In several South African SOC studies, the prevalence of confirmed virologic failure ranged from 4 to 33% based on varying times on ART, different definitions of confirmed virologic failure and first‐ or second‐line ART regimens [23, 24, 25, 26]. ACs, a client‐centred approach, focuses on the dynamic needs of patients by offering convenient ART services with the potential benefit of increasing adherence and retention. Our study suggests that PLHIV who have been categorized as stable in SOC can be safely transitioned to ACs after scale‐up, thus creating space in the SOC for high‐risk patients who require targeted and specific interventions.

VL monitoring is critical in reducing HIV‐related mortality and increasing early detection of virologic failure, and has become a proxy for the quality of care and functioning of ACs [27, 28]. We found that patients were generally monitored consistently over time. More than 80% of patients had VLs done at the four time periods assessed during the study period, similar to the proportion of VLs done in Khayelitsha, suggesting that AC patients are receiving comparable care [29]. Of those tested, over 90% of patients remained virologically suppressed, consistent with previous findings [3]. This positive finding provides evidence of the robustness of ACs in the context of rapidly expanding ART services.

However, we observed deviations from AC guidelines, which may have impacted on patient outcomes. First, approximately 10% of patients never received a VL test after AC entry, highlighting concerns about inadequate monitoring of patients [18]. Second, repeat VL testing was suboptimal among patients with an elevated VL. Approximately one third of these patients did not receive a repeat test within one year of experiencing an elevated VL and approximately a quarter of these patients never received a VL test after experiencing an elevated VL. As a result, we could have underestimated the true proportion of virologic failure over time. Compliance to AC guidelines is critical for favourable patient outcomes and VL monitoring should be used as a key monitoring tool for patients, with a targeted and sustained approach to VL monitoring for high‐risk patients even within DSDs.

In more recent years, the risk of experiencing an elevated VL was reduced despite the scale‐up of ACs. We do not believe year of AC entry was acting as a confounder, because this decreased risk was observed even when we restricted the analysis for the same duration of follow‐up time. However, patients who experienced an elevated VL in more recent years were at higher risk of experiencing confirmed virologic failure, suggesting that high‐risk patients may not have received appropriate interventions in more recent years, possibly due to AC scale‐up. Duration on ART at AC entry had little effect on the estimates. However, in the sensitivity analysis, we found an increased risk for confirmed virologic failure, similar to findings exploring viral rebound in ACs [3].

Older patients within ACs had a decreased risk of experiencing an elevated VL, a finding consistent with previous studies which used different age thresholds [3, 24, 30]. The increased risk of poor virologic outcomes in younger patients may be due to these patients having more difficulty transitioning between models of care or that the current AC model being implemented within this setting is not actually tailored to meet their needs. Younger patients constitute a large percentage of new HIV infections and retaining these patients in care is challenging as they have to deal with a lifelong chronic illness and adhering to treatment, while increasing their autonomy over health‐related decisions [31, 32, 33, 34]. As ACs mature, it is important to understand the specific vulnerabilities of younger patients and to introduce ACs that meet their needs.

In our study, we found no sex difference in those experiencing either an elevated VL or confirmed virologic failure. This is consistent with other studies which observed no sex differences in virologic failure, LTFU, attrition and virologic rebound rates within SOC or DSD models [3, 23, 30, 35]. However, several South African studies have reported an increased risk of virologic failure among men [24, 25, 26, 36]. Given the growing body of evidence that men are disadvantaged in access to, and outcomes on ART [37, 38, 39, 40, 41, 42, 43, 44, 45], our findings suggest that ACs may have a particular benefit for men who chose to be in one, and that stable men in routine care could be safely referred to ACs for ongoing care. This model of care appears to be beneficial for women as well, given that 74% of the AC cohort were women, approximately 10% higher than the proportion of HIV‐positive women in South Africa [46].

This study is strengthened by the duration of follow‐up after the scale‐up of ACs, high retention rates, the large sample size and the exploration of virologic failure after AC entry, which to our knowledge has not been analysed previously. There are several limitations to the interpretation of these results. The low prevalence of confirmed virologic failure should be interpreted with caution due to missing VLs after AC entry, especially as more patients entered ACs in more recent years. However, we excluded patients entering in 2017, allowing 12 months for a VL test to be done as per the guidelines. Although data were analysed for several years, the median follow‐up time was relatively short as the majority of patients entered ACs in more recent years. Also, key variables such as the date of AC exit, the use of a buddy, ART regimen and adherence were not available to be assessed. We did however have the last clinical visit date, allowing us to determine retention, LTFU and transfer out. At the time of this visit, these patients could have been in ACs or receiving clinical care. Lastly, this analysis included adult patients from Khayelitsha, a peri‐urban setting, limiting the generalizability of the study. Patients in ACs were generally older, predominantly female, had higher CD4 counts and a longer median duration on ART at AC entry than the general population on ART. This suggests that our findings may be generalizable to stable patients entering ACs, but not necessarily to all patients on ART.

As the number of people requiring lifelong ART increases, the need for DSD models will grow. ACs are increasingly seen as an alternate method to improve clinical outcomes in burdened healthcare settings, with the flexibility, acceptability and implementation of the programme strongly contributing to its sustainability [47]. Additionally, patients have reported improved satisfaction with the convenience of ACs [28, 48]. Pilot projects of ACs are underway in the Democratic Republic of Congo and Zambia [49], with national policies in Kenya, Zimbabwe and Swaziland supporting the scale‐up of modified AC models [49, 50, 51, 52]. Locally, ACs have been successfully scaled up in the Cape Metro Health District, adopted into national guidelines and implemented in several provinces [7, 9, 10]. Our study provides additional evidence that ACs offer an effective option for long‐term chronic care for stable patients in this setting. However, more data are required to understand these patients’ experiences, and an updated cost‐effectiveness analysis would be beneficial to policy makers.

## 5 Conclusions

In summary, we observed good long‐term virologic responses to ART in patients accessing ART within ACs. High rates of retention, VL testing and virologic suppression were maintained throughout the study period. AC eligibility and VL monitoring guidelines should be strictly adhered to, especially among high‐risk patients. These findings support the continued scale‐up of ACs as an alternative for ART delivery for stable patients in the national ART programme in South Africa.

## Competing interests

The authors have no conflicts of interest to declare.

## Authors’ contributions

KK, JE and MC conceptualized the research study. PRT, JE and MC assisted with the data management. KK was responsible for data cleaning, statistical analyses and the initial draft manuscript. All authors (KK, AB, PRT, JE, MAD and MC) reviewed, revised and approved the final manuscript.

## Supporting information


**Table S1** **.** Among patients who were virologically suppressed six months before adherence club entry, crude and adjusted associations with an elevated viral load and confirmed virologic failure, after entry into an adherence club.
**Table S2** **.** Among patients who were virologically suppressed 15 months before adherence club entry and followed up for 475 days, crude and adjusted associations with an elevated viral load and confirmed virologic failure, after entry into an adherence club.
