# Supplementary material for: Long‐term virologic responses to antiretroviral therapy among HIV‐positive patients entering adherence clubs in Khayelitsha, Cape Town, South Africa: a longitudinal analysis
Source: J Int AIDS Soc. 2020 May 14;23(5):e25476. doi: 10.1002/jia2.25476 (PMC7224308; doi:10.1002/jia2.25476)
Supplement: Supplementary file 1 — Table S1 . Among patients who were virologically suppressed six months before adherence club entry, crude and adjusted associations with an elevated viral load and confirmed virologic failure, after entry into an adherence club. Table S2 . Among patients who were virologically suppressed 15 months before adherence club entry and followed up for 475 days, crude and adjusted associations with an elevated viral load and confirmed virologic failure, after entry into an adherence club. [file JIA2-23-e25476-s001.docx]

**Supplementary Table 1. Among patients who were virologically suppressed six months before adherence club entry, crude and adjusted associations with an elevated viral load and confirmed virologic failure, after entry into an adherence club.**

|  | **First elevated viral load** | | **Confirmed virologic failure** | |
| --- | --- | --- | --- | --- |
|  | **Model 1 HR (95% CI)** | **Model 2 aHR (95% CI)**  **(n = 2 863)** | **Model 1 HR (95% CI)** | **Model 2 aHR (95% CI)**  **(n = 116)** |
| **Sex** |  |  |  |  |
| Male | 0.96 (0.71-1.31) | 1.09 (0.74-1.58) | 0.75 (0.41-1.36) | 1.18 (0.58-2.39) |
| **Age group (years)** |  |  |  |  |
| 16-34 | 1.0 | 1.0 | 1.0 | 1.0 |
| 35-44 | 0.68 (0.51-0.92) | 0.52 (0.37-0.74) | 0.97 (0.60-1.58) | 0.73 (0.40-1.34) |
| ≥45 | 0.64 (0.44-0.93) | 0.52 (0.34-0.81) | 0.59 (0.30-1.16) | 0.34 (0.15-0.81) |
|  |  |  |  |  |
| **Year of AC entry** | 0.82 (0.72-0.93) | 0.75 (0.65-0.87) | 1.39 (1.10-1.76) | 1.12 (0.83-1.52) |
| **Duration on ART at AC entry (years)** |  |  |  |  |
| 0-2 | 1.0 | 1.0 | 1.0 | 1.0 |
| 3-4 | 1.30 (0.88-1.91) | 1.22 (0.78-1.92) | 1.15 (0.56-2.34) | 1.15 (0.47-2.80) |
| ≥5 | 1.17 (0.82-1.67) | 1.48 (0.97-2.24) | 1.69 (0.90-3.20) | 2.53 (1.14-5.60) |
| **CD4 count at AC entry(cells/µL)** |  |  |  |  |
| <500 | 1.0 | 1.0 | 1.0 | 1.0 |
| ≥500 | 0.79 (0.58-1.07) | 0.76 (0.59-1.08) | 0.74 (0.44-1.24) | 0.61 (0.35-1.06) |

These models used six months before AC entry to determine if patients were suppressed.

Model 1: univariable analysis; model 2: multivariable analysis including sex, age, year of AC entry, duration on ART at AC entry and CD4 count at AC entry.

For each outcome, only patients with a viral load on file were included in the model.

**Supplementary Table 2. Among patients who were virologically suppressed 15 months before adherence club entry and followed up for 475 days, crude and adjusted associations with an elevated viral load and confirmed virologic failure, after entry into an adherence club.**

|  | **First elevated viral load** | | **Confirmed virologic failure** | |
| --- | --- | --- | --- | --- |
|  | **Model 1 HR (95% CI)** | **Model 2 aHR (95% CI)**  **(n = 4 590)** | **Model 1 HR (95% CI)** | **Model 2 aHR (95% CI)**  **(n = 208)** |
| **Sex** |  |  |  |  |
| Male | 0.91 (0.63-1.32) | 1.0 (0.68-1.46) | 0.48 (0.19-1.24) | 0.56 (0.19-1.68) |
| **Age group (years)** |  |  |  |  |
| 16-34 | 1.0 | 1.0 | 1.0 | 1.0 |
| 35-44 | 0.83 (0.58-1.18) | 0.81 (0.56-1.17) | 1.44 (0.74-2.80) | 1.13 (0.53-2.44) |
| ≥45 | 0.64 (0.41-1.02) | 0.63 (0.39-1.01) | 0.28 (0.06-1.23) | 0.15 (0.02-1.20) |
|  |  |  |  |  |
| **Year of AC entry** | 0.73 (0.63-0.84) | 0.73 (0.63-0.84) | 0.93 (0.70-1.23) | 0.74 (0.51-1.07) |
| **Duration on ART at AC entry (years)** |  |  |  |  |
| 0-2 | 1.0 | 1.0 | 1.0 | 1.0 |
| 3-4 | 1.16 (0.74-1.84) | 1.13 (0.71-1.79) | 0.73 (0.27-1.97) | 0.50 (0.16-1.56) |
| ≥5 | 1.07 (0.70-1.63) | 1.18 (0.77-1.81) | 1.13 (0.48-2.63) | 1.13 (0.44-2.92) |
| **CD4 count at AC entry(cells/µL)** |  |  |  |  |
| <500 | 1.0 | * | 1.0 | 1.0 |
| ≥500 | 0.51 (0.34-0.74) | - | 0.84 (0.40-1.75) | 0.73 (0.35-1.54) |

These models were restricted to a follow-up time of 475 days (the sum of the median times to an elevated viral load and confirmed virologic failure).

Model 1: univariable analysis; model 2: multivariable analysis including sex, age, year of AC entry, duration on ART at AC entry and CD4 count at AC entry.

For each outcome, only patients with a viral load on file were included in the model.

*Log likelihood cannot be estimated
